# Adaptive Access Selection Algorithm for Large-Scale Satellite Networks Based on Dynamic Domain

**DOI:** 10.3390/s22165995

**Published:** 2022-08-11

**Authors:** Gaosai Liu, Xinglong Jiang, Huawang Li, Zhenhua Zhang, Siyue Sun, Guang Liang

**Affiliations:** 1 Innovation Academy for Microsatellites of CAS, Shanghai 201204, China; 2 University of Chinese Academy of Sciences, Beijing 100049, China; 3 Shanghai Engineering Center for Microsatellites, Shanghai 201204, China

**Keywords:** large-scale satellite network, access selection algorithm, satellite access technology, resource allocation, StarLink

## Abstract

The traditional satellite access selection algorithm, which is used in large-scale satellite networks, has some disadvantages, such as frequent link switching, high interrupt probability, and unable to adapt to a dynamic environment. According to the periodicity of the large-scale satellite network and the prior knowledge provided by acknowledgment packages, a dynamic domain-based adaptive access algorithm (DAA) is proposed in this paper. Firstly, this algorithm divides the large-scale satellite network into different domains according to the minimum elevation angle of the Earth station (ES) and the predictable characteristics of the trajectory of the satellite. Then, the ES selects the access satellites according to the relationship between the traffic volume and the satellites’ coverage time. Finally, the ES selects the backup access satellite based on the satellites’ coverage time, the traffic volume of the ES, satellite status provided by prior knowledge, and other information. When the access satellite cannot satisfy the communication demand, the ES adaptively switches the earth-satellite link to the backup access satellite. The ES first choice of access satellite does not require interaction with the satellites, reducing the consumption of communication resources. The selection strategy of backup access satellite and the concept of virtual destination address proposed in this paper can reduce the routing overhead after switching. Through theoretical analysis and simulation results in the StarLink constellation, it is proved that this paper improves the coverage time utilization of accessing satellites and reduces the switching probability compared with the traditional access algorithm, which is more suitable for ES to access large-scale satellite networks.

## 1. Introduction

A large-scale satellite network has the characteristics of low propagation delay, high-dynamic, and multiple satellites that can be selectively accessed by the Earth station (ES). It has received more attention in constructing 5/6G and the Internet of Things (IoT) [1,2]. In recent years, satellite Internet has ushered in a new wave of development. To satisfy the demand for high-capacity communication from ES, satellite Internet mainly uses large-scale satellite constellations. Typical large-scale constellations include the StarLink, the OneWeb, and the Amazon Kuiper. The SpaceX designed StarLink constellation is expected to include approximately 12,000 satellites in the low earth orbit and very low earth orbit constellations [3]. The first phase of the StarLink constellation consists of 4409 satellites, distributed in five different altitude Shells. The satellites on the same shell are at the same altitude from the ground. The Kuiper constellation plan consists of 3236 satellites [4]. The OneWeb constellation plan consists of 650 satellites [5]. Large-scale satellite networks can provide broadband access and high-speed Internet services in theory. However, the rapid topology changes of large-scale satellite networks, the limited resources of a single satellite, and the high switching frequency of earth-satellite links (ESLs) have brought new challenges to the access selection of satellite networks.

Access selection is the primary link for ES to transmit data using a satellite network. The state of access satellite affects the quality of services (QoS), such as transmission delay, outage probability, and call blocking rate. Traditional access selection strategies include single parameter-based access selection algorithms and multi-attribute-based access selection algorithms. Access selection algorithms based on a single parameter include the longest coverage time access algorithm, the shortest transmission distance access algorithm, and load balancing algorithm [6,7,8,9,10,11,12]. These methods mainly select access satellites based on a single target attribute. For example, in Literature [6], to reduce the switching times, the access strategy is proposed with the goal of the longest coverage time. In Literature [10], to reduce the path loss and improve the signal-to-noise ratio, the access strategy was proposed with the shortest transmission distance as the goal. In Literature [11] is to achieve network load balancing. The ES will choose the satellite which is beneficial to the network load balancing as the access satellite. The most widely used access algorithm based on multiple attributes is the comprehensive weighting algorithm [13,14,15,16,17,18]. For example, the weighted algorithm based on QoS proposed in Literature [17]. The algorithm chooses the access satellite after weighting the shadow effect, signal-to-noise ratio, and elevation angle. This method effectively reduces the call blocking rate and switching failure rate. Based on the comprehensive linear weighted multi-satellite coverage access selection strategy is proposed in [18]. The elevation angle, the distance between the ES and the satellite, the coverage time, and the number of idle channels are comprehensively weighted, which reduces the blocking rate and the outage probability of ESL.

The combined free demand assignment multiple access Protocol (CFDAMA) has received much attention from researchers [19,20,21]. Free access is assigned by polling, and on-demand access is assigned by reservation [22]. To improve the access performance of CFDAMA, researchers have improved CFDAMA from different perspectives. For example, in literature [19], to realize the data transmission of the ESs in time, the coverage time of the satellite is fully utilized. A combined free/demand assignment multiple access schemes based on convolutional long short term memory network and transfer learning (CFDAMA-CLSTMTL) is proposed by periodically predicting the data generated by ESs. The simulation results show that CFDAMA—CLSTMTL reduces the end-to-end delay. In Literature [21], a combined free/demand assignment multiple access schemes employing a combined round robin/piggybacking request strategy (CFDAMA-RPR) is proposed to improve the throughput and reduce the end-to-end delay of satellite networks. The improved algorithm based on CFDAMA improves the network throughput to a certain extent and reduces the end-to-end delay. However, implementing these algorithms requires the interaction between satellites and ESs, increasing the cost of network resources. In fact, simple processing of acknowledgment packages (ACKs) can somewhat reduce the number of interactions between satellites and ES.

The above access selection algorithm reduces the switching failure rate, blocking rate, and outage probability to some extent. However, the multi-satellite coverage of the above algorithm is mostly small constellations, the inter-satellite collaboration is not considered when selecting the access satellite, and the characteristics of satellite networks such as predictable trajectory are not fully utilized. Because of the shortcomings of existing access selection algorithms in large-scale satellite networks, we propose a dynamic domain-based adaptive access algorithm (DAA). The main contributions of the DAA algorithm proposed in this paper are as follows:

- The strategy of large-scale satellite network dynamic domain is proposed. Based on the predictable characteristics of satellite position, the ES determines the communication domain according to the minimum elevation angle, and the satellites in the communication domain change dynamically with time. Before the ES sends data packets, it calculates each satellite’s position information and coverage time in the communication domain through the stored ephemeris information. The calculation and storage costs of this process are all from the ES, and there is no need for information interaction between satellites and the ES, reducing resource-constrained satellites’ storage and calculation costs.
- This paper proposes an adaptive access selection algorithm based on the dynamic domain. Firstly, the ES determines access satellites according to the relationship between satellite coverage time and ES traffic volume. Then the ES determines the backup access satellite according to the prior knowledge. ES can quickly switch to a backup satellite when switching access satellite is required.
- To reduce the routing overhead caused by switching the ESL to the backup access satellite, the DAA algorithm preferentially selects backup access satellites in the original path. When there is no satellite in the original path to meet the access requirements, this paper proposes the concept of a virtual destination address. The backup access satellite first transmits the data packet to the virtual destination address. Then the satellite corresponding to the virtual destination address transmits the data packet to the destination node.
- To verify the effectiveness of the DAA algorithm, we model and analyze the access selection of the StarLink constellation. Modeling and analysis methods provide a reference for subsequent research on large-scale constellations.

The organization of the remainder of this article is as follows: Section 2 describes the system model and defines the problems such as communication domain and coverage time. Section 3 describes the specific implementation of the DAA algorithm. Section 4 simulates and analyzes the performance of the DAA algorithm. Finally, Section 5 summarizes this article.

## 2. System Model and Problem Definition

### 2.1. Large-Scale Satellite Network Model

**Figure 1:**
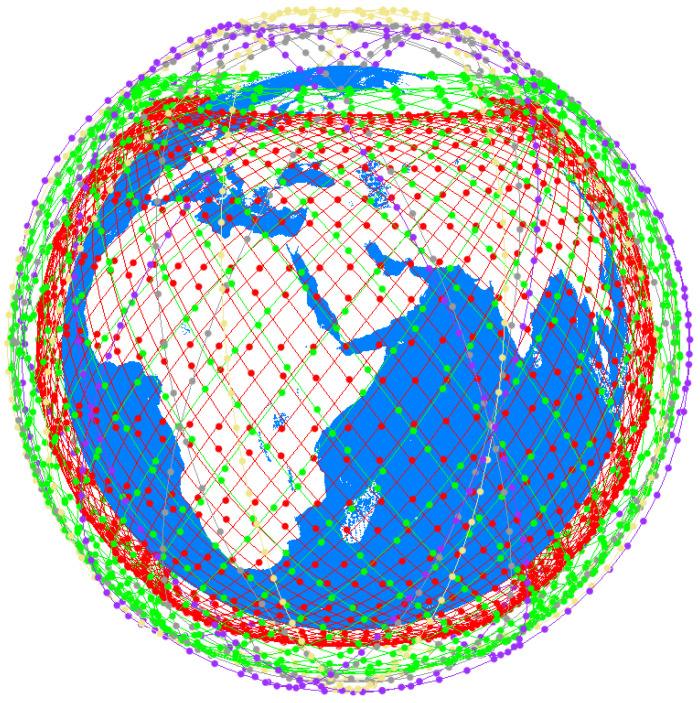
The first phase of Starlink.

This paper uses the large-scale constellation StarLink as the modeling object. The first phase of the Starlink constellation consists of five Shells [23] defined as $S^{1}$, $S^{2}$, $S^{3}$, $S^{4}$, and $S^{5}$ from near to far from the ground. The number of orbits of each Shell, the number of satellites in each orbit, and the orbital inclination parameters are shown in Table 1. Figure 1 shows the schematic diagram of the StarLink constellation. Each color represents a shell.

The constellation is defined as an undirected graph $G=\left(S,E\right)$, where S represents the satellite nodes, defined as $S=\left\{S_{1,1}^{1},S_{1,2}^{1},\ldots ,S_{n,m}^{L},\ldots ,S_{N_{5},M_{5}}^{5}\right\}$, where *L* denotes the Shell serial number, *n* denotes the orbit serial number, *m* denotes the satellite serial number within the orbit, $N_{L}$ denotes the total number of orbits on shell $S^{L}$, $M_{L}$ denotes the number of satellites per orbit of shell $S^{L}$. Table 1 shows that there are $\sum_{L=1}^{5}N_{L}\times M_{L}=4409$ satellites in StarLink constellation. E represents the set of inter-satellite links (ISLs) and ESLs in the constellation, $E=\left[ISL,ESL\right]$. Each satellite can establish ISLs with the surrounding satellites [24], and satellites in the communication domain of ES can establish ESLs.

**Figure 2:**
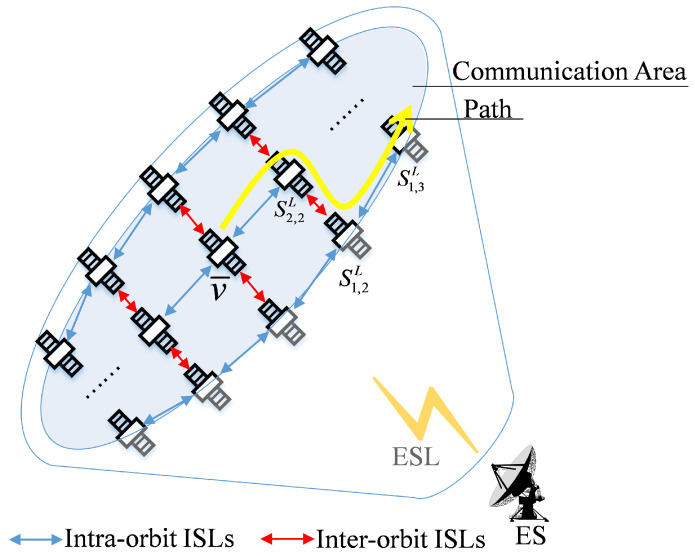
Communication domain of the ES.

Figure 2 shows the topological relationship between the ES and the satellite network. The shadow area in the figure is the communication domain calculated according to the minimum elevation angle of the ES. Define the set of satellites in the communication domain of the ES as V. If $S_{n,m}^{L}\in V$, then $S_{n,m}^{L}$ can establish an ESL with ES. The satellite establishing ESL with ES is defined as $\bar{v}$. ES sends packets to $\bar{v}$, and the satellite forwards packets according to the routing table. As the yellow arrow in Figure 2 shows the packet forwarding path, the set of satellites on the path is defined as $V^{\prime }$. The satellite set in the communication domain and belonging to the forwarding path is defined as $V^{\prime \prime }$, namely $V^{\prime \prime }=V\cap V^{\prime }$. In Figure 2, $\left\{\bar{v},S_{2,2}^{L},S_{1,2}^{L},S_{1,3}^{L}\right\}\subseteq V^{\prime \prime }$.

### 2.2. Problem Definition

**Figure 3:**
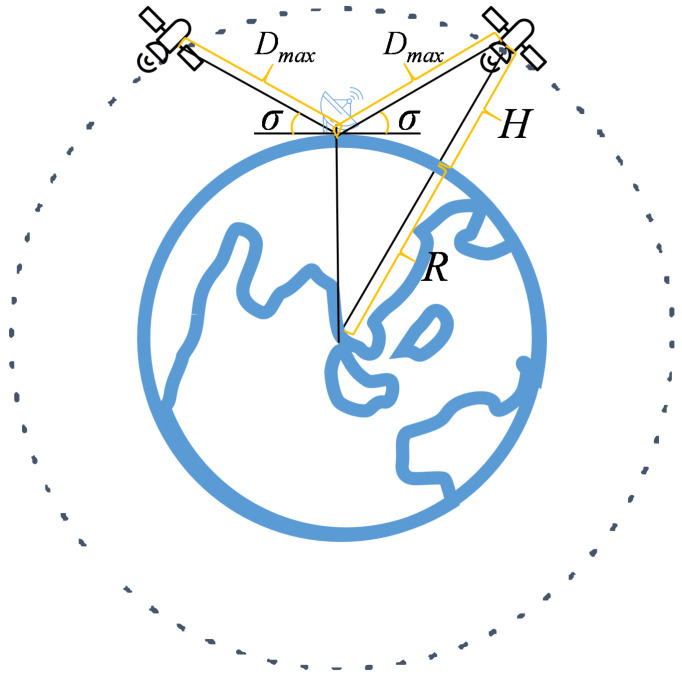
Parameters in Equation (1).

ES needs to determine the communication domain before sending packets. According to the minimum elevation angle of ES, the maximum communication distance of ES is calculated as:

(1) $$\begin{array}{c} D_{\max}=\sqrt{{\left(R+H\right)}^{2}+R^{2}-2\left(R+H\right)R\cos\left(\alpha \right)}\\ \alpha =90^{o}-\sigma -\arcsin\left(\frac{R}{R+H}\sin\left(R+H\right)\right), \end{array}$$

where *R* is the radius of the Earth, *H* is the distance from the satellite to the ground, σ is the minimum elevation of ES, and α is a temporary variable. The meaning of parameters in Equation (1) is shown in Figure 3.

ES can predict the position of the satellites in real-time according to the orbital elements [25]. The latitude and longitude of $S_{n,m}^{L}$ at time *t* are defined as $LOC_{L,n,m}^{t}$:

(2) $$\begin{array}{c} LOC_{L,n,m}^{t}=\left[LON_{L,n,m}^{t},LAT_{L,n,m}^{t}\right]\\ \left[LON_{L,n,m}^{t},LAT_{L,n,m}^{t}\right]=P_{calc}\left(i,\Omega ,e,\omega ,a,M_{0},t\right), \end{array}$$

where $i,\Omega ,e,\omega ,a,M_{0}$ is orbital elements, *t* is the time when ES calculates the longitude and latitude of the satellite. The value of *t* can be the current time or the future time. $P_{calc}$ is a function of calculating the longitude and latitude of the satellite at time *t* by orbital elements. According to the maximum communication distance of ES and the longitude and latitude of the satellite at *t* time, the coverage time $t_{L,n,m}^{con}$ of $S_{n,m}^{L}$ is calculated:

(3) $$\begin{array}{c} D_{ES,t}^{L,n,m}=f\left(LON_{ES},LAT_{ES},H_{ES},LON_{L,n,m}^{t},LAT_{L,n,m}^{t},H_{L}\right)\\ LOC_{L,n,m}^{out}=\underset{LON,LAT}{argD_{\max}}\left(D_{ES,t}^{L,n,m}\right)\\ t_{L,n,m}^{out}=\underset{t}{argLOC_{L,n,m}^{out}}\left(P_{calc}\right)\\ t_{L,n,m}^{con}=t_{L,n,m}^{out}-t_{0}, \end{array}$$

where $D_{ES,t}^{L,n,m}$ is the linear distance from $S_{n,m}^{L}$ to ES. $LON_{ES}$, $LAT_{ES}$, and $H_{ES}$ are the longitude, latitude, and altitude of the ES, respectively. $H_{L}$ is the distance from $S_{n,m}^{L}$ to the ground, and *f* is the function to calculate the distance between ES and $S_{n,m}^{L}$. arg is the deformation of the inverse function. For example, $Z=\underset{t}{argX}\left(f(t)\right)$ denotes that when f(t)=X, the corresponding *t* value is assigned to *Z*. A similar function to arg is used in Literature [26]. $LOC_{L,n,m}^{out}$ denotes the latitude and longitude of $S_{n,m}^{L}$ when $D_{ES,t}^{L,n,m}$ equals $D_{\max}$. $t_{L,n,m}^{out}$ denotes the corresponding time when $P_{calc}$ equals $LOC_{L,n,m}^{out}$. $t_{0}$ is the current moment.

Satellites can obtain their latitude, longitude, and altitude in real-time through the positioning system. From Equations (1) and (3), when $D_{ES,t}^{L,n,m}\leq D_{\max}$, $S_{n,m}^{L}\in V$, $S_{n,m}^{L}$ is in the communication domain of ES.

The traditional maximum coverage time algorithm and maximum elevation angle algorithm select the accessing satellites according to the coverage time and elevation angle, respectively, without considering the different demands of different ES for communication time at different times, which will cause problems such as wasting resources and increasing the number of switching. ES needs to send *k* data packets at time *t*, the size of the *i* packet is denoted as $pkg_{i}$, and the size of the last ACK is $pkg_{A}$. The uplink and downlink of StarLink use different channels [27]. The uplink transmission rate is defined as $R_{U}$, and the downlink transmission rate is defined as $R_{D}$. The minimum time that ES needs to occupy ESL is $t_{u}$:

(4) $$t_{u}=\frac{\sum_{i=0}^{k-1}pkg_{i}}{R_{U}}+\frac{pkg_{A}}{R_{D}}.$$

The coverage time utilization rate of access satellite is an important parameter to measure the access algorithm. A higher utilization rate indicates that the access satellites selected are better matched with $t_{u}$. The utilization rate of access satellite coverage time is defined as $P_{u}$:

(5) $$P_{u}=\frac{t_{u}}{\sum_{L,n,m}^{Q}t_{L,n,m}^{con}},$$

where Q denotes the set of access satellites. $\sum_{L,n,m}^{Q}t_{L,n,m}^{con}$ represents the sum of coverage time of all satellites in Q from time $t_{0}$. To achieve the maximum $P_{u}$ value, the ES prefers to access satellite $\bar{v}$:

(6) $$\begin{array}{cc} \bar{v}=\underset{S}{arg\min}\left(t_{V}^{con}\right) & t_{L,n,m}^{con}\geq t_{u}, \end{array}S_{n,m}^{L}\in V,$$

where $t_{V}^{con}$ is the set of satellite coverage times that satisfy $t_{L,n,m}^{con}\geq t_{u}$ in the ES communication domain. $\bar{v}$ is the satellite corresponding to the minimum in set $t_{V}^{con}$.

**Figure 4:**
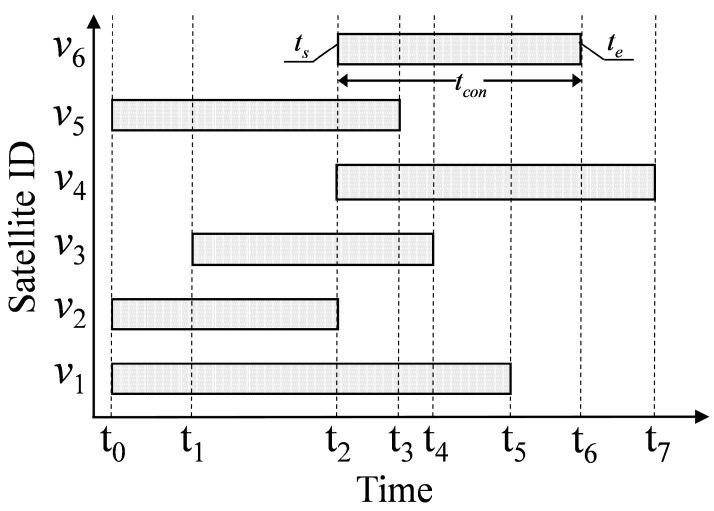
Satellites coverage time at different moments.

The satellite coverage time in the communication domain of the ES is schematically shown in Figure 4. $t_{s}$ and $t_{e}$ are the start and end moments of the coverage time, respectively. Select the access satellite according to Equation (6). If ES is at time $t_{0}$, the traffic volume to be transmitted is $t_{u}$. When $t_{u}<\left(t_{2}-t_{0}\right)$, ES preferentially chooses $v_{2}$ as the access satellite. When $\left(t_{2}-t_{0}\right)<t_{u}<\left(t_{3}-t_{0}\right)$, ES preferentially chooses $v_{5}$ as the access satellite. When $\left(t_{4}-t_{0}\right)<t_{u}<\left(t_{5}-t_{0}\right)$, ES preferentially chooses $v_{1}$ as the access satellite. The access satellites selected above can theoretically complete data transmission without switching ESLs. However, when $t_{u}$ is larger than the coverage time of any satellite, it is necessary to switch the access satellite. In this case, how to choose the access satellite set Q and the access sequence to maximize the $P_{u}$ value. We will give a strategy to solve this problem in the next section.

## 3. DAA Algorithm Design

### 3.1. Access Selection Mechanism

The DAA algorithm calculates the first time access satellite of ES through the following steps.

1. The communication domain of ES is calculated by Equation (1);
2. ES calculates which satellites in the constellation are located in the communication domain by Equation (2).
3. ES calculates the satellite coverage time in the communication domain by Equation (3).
4. ES compares the relationship between the satellites’ coverage time and $t_{u}$. If there is a satellite covering time greater than $t_{u}$, then $t_{V}^{con}\neq \emptyset$ in Equation (6). The first time access satellite is calculated directly through Equation (6). If the coverage time of all satellites is less than $t_{u}$, then $t_{V}^{con}=\emptyset$ in Equation (6). The access satellite of ES cannot be calculated by Equation (6).

Next, we will introduce the access satellite selection strategy of the DAA algorithm when $t_{V}^{con}=\emptyset$.

**Figure 5:**
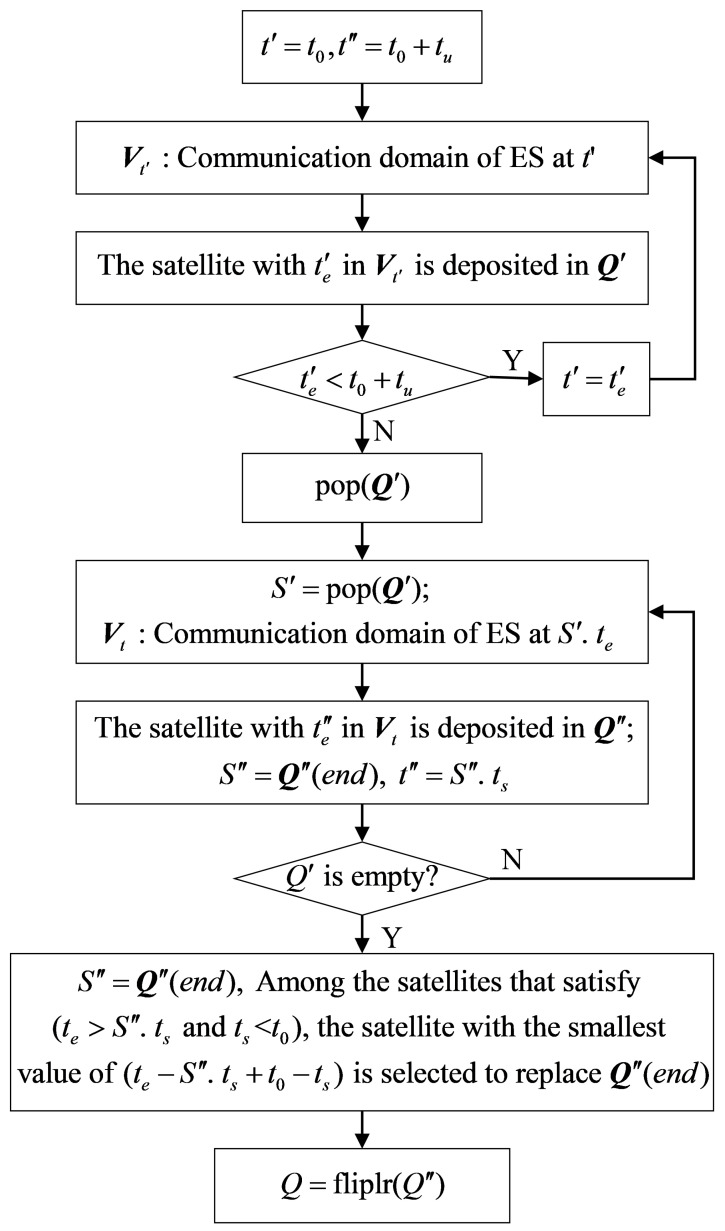
Flow chart of the algorithm to calculate the Q.

As shown in Figure 4, if the traffic volume transmitted by the ES at time $t_{1}$ is $t_{u}$. When $\left(t_{6}-t_{1}\right)>t_{u}>\left(t_{5}-t_{1}\right)$, then $t_{V}^{con}=\emptyset$. Choosing any satellite cannot complete the data transmission without switching ESL. To solve the access problem for $t_{V}^{con}=\emptyset$ and to achieve the minimum number of switching and maximum $P_{u}$ value. This paper defines the set of ES’s access satellites at time *t* as Q. The satellites are stored in Q according to the access sequence of ES. The flow chart of calculation Q is shown in Figure 5.

In Figure 5, $t^{\prime }$ and $t^{\prime \prime }$ are the time variables. $Q^{\prime }$ and $Q^{\prime \prime }$ are the set variables. pop($Q^{\prime }$) denotes removing the last element from $Q^{\prime }$. $Q^{\prime \prime }$(end) represents the last element in $Q^{\prime \prime }$. $t_{e}^{\prime }$ is the end moment of the maximum coverage time of the satellites in $V_{t^{\prime }}$. $t_{e}^{\prime \prime }$ is the minimum positive value obtained by subtracting $t^{\prime \prime }$ from the end time of satellite coverage in $V_{t}$. $S^{\prime }$ and $S^{\prime \prime }$ are satellite variables. $S^{\prime }\cdot t_{e}$ denotes the end moment of $S^{\prime }$ coverage and $S^{\prime \prime }\cdot t_{s}$ denotes the start moment of $S^{\prime \prime }$ coverage. Q = fliplr $\left(Q^{\prime \prime }\right)$ denotes that the elements in the set $Q^{\prime \prime }$ are assigned to Q after reverse order.

In Figure 4, the access satellite set Q of the ES at time $t_{1}$ is calculated according to the above process. When $t_{u}$ satisfies $\left(t_{5}-t_{1}\right)<t_{u}\leq \left(t_{6}-t_{1}\right)$, the set of access satellites is $Q=\{v_{2},v_{6}\}$.

### 3.2. Access Switching Mechanism

When the coverage time of a single satellite is not enough to transmit data from an ES, it is necessary to switch access to a new satellite. An ES also needs to switch ESL to a new access satellite in the following cases:

1. The remaining resources of $\bar{v}$ are below the threshold, such as memory capacity and energy. The ES can receive the ACKs correctly for this case;
2. ESL interruption or access satellite failure. In this case, ES cannot receive the ACKs.

#### 3.2.1. Insufficient Satellite Resources

To solve the problem of insufficient access satellite resources, the ES selects the backup access satellite ${\bar{v}}^{\prime }$ according to the prior knowledge obtained from the ACKs and the coverage time of the satellites. To obtain the state information of some satellites in the communication domain without the additional interactive information between the satellite and the Earth Station, the DAA algorithm does the following processing:

1. When ES sends data packets to the access satellite, $\left[LON_{ES},LAT_{ES},D_{\max}\right]$ is added to the data packets;
2. After receiving the data packets sent by the ES, the access satellite forwards the data packets according to the routing table. After the satellite $S_{n,m}^{L}$ in the set $V^{\prime }$ receives the data packet, it decodes $\left[LON_{ES},LAT_{ES},D_{\max}\right]$ first, and then determines whether it is in the communication domain of the ES by Equation (3). If $t_{L,n,m}^{con}>0$ in Equation (3), $S_{n,m}^{L}$ is located in the communication domain of the ES.
3. If $S_{n,m}^{L}$ is in the communication domain of the ES, $\left[C_{L,n,m}^{t},E_{L,n,m}^{t}\right]$ is added to the ACKs when $S_{n,m}^{L}$ transmits ACKs. $C_{L,n,m}^{t}$ represents the residual memory capacity of $S_{n,m}^{L}$ at time *t*, and $E_{L,n,m}^{t}$ represents the residual energy of $S_{n,m}^{L}$ at time *t*.
4. If $S_{n,m}^{L}$ is not in the communication domain of the ES, $S_{n,m}^{L}$ will delete [$LON_{ES}$, $LAT_{ES}$, $D_{\max}$] before forwarding the data packet. $S_{n,m}^{L}$ does not extra process the ACKs.

**Figure 6:**
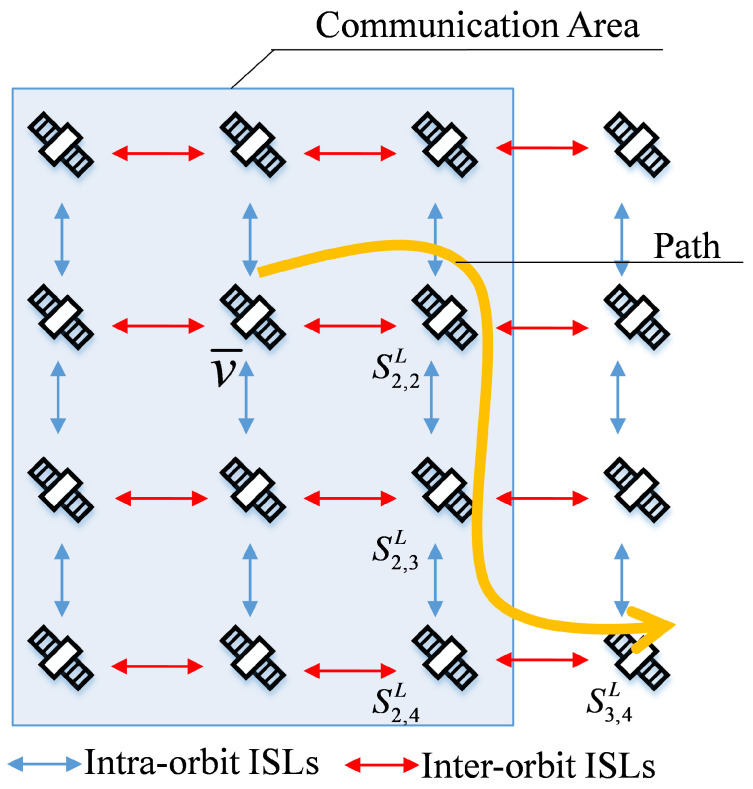
The relationship between packet transmission path and communication domain Equation (1).

**Figure 7:**
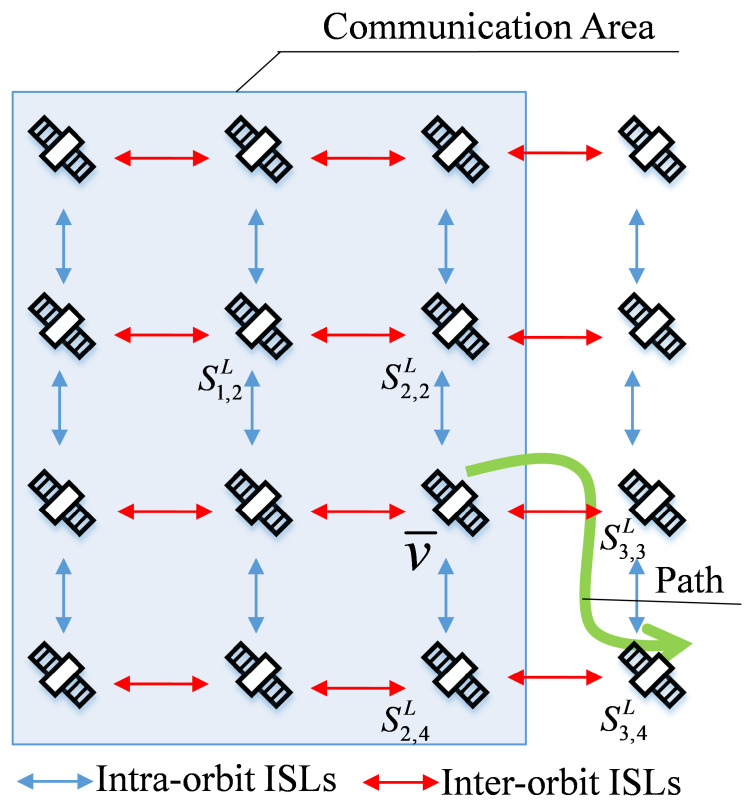
The relationship between packet transmission path and communication domain Equation (2).

Through the above steps, ES can obtain prior knowledge of the satellites in the set $V^{\prime \prime }$. Set $V^{\prime \prime }$ in some scenes includes multiple satellites. As shown in Figure 6, the set $V^{\prime \prime }$ includes $S_{2,2}^{L}$, $S_{2,3}^{L}$ and $S_{2,4}^{L}$ in addition to access satellite $\bar{v}$. However, in some scenarios, set $V^{\prime \prime }$ only includes access satellites $\bar{v}$. As shown in Figure 7, set $V^{\prime \prime }$ only includes $\bar{v}$.

For the scenario in Figure 6, the ES receives the memory capacity and energy remaining state of the satellites in $V^{\prime \prime }$ through ACKs. Calculate memory capacity and energy surplus rate according to the following Equations:

(7) $$U_{C}=\frac{C_{L,n,m}^{t}}{C_{L,n,m}},U_{E}=\frac{E_{L,n,m}^{t}}{E_{L,n,m}},$$

where $C_{L,n,m}$ and $E_{L,n,m}$ represent the maximum memory capacity and the maximum energy value of $S_{n,m}^{L}$, respectively. $U_{C}^{T}$ and $U_{E}^{T}$ represent the residual memory and energy capacity thresholds, respectively. The ES first calculates the coverage time of the satellites in set $V^{\prime \prime }$ by Equation (3), then selects the satellite with $U_{C}$, $U_{E}$ and coverage time satisfying the traffic volume requirements as backup access satellite ${\bar{v}}^{\prime }$. If there are multiple satellites in set $V^{\prime \prime }$ satisfying the condition of ${\bar{v}}^{\prime }$, one of them is selected as the ${\bar{v}}^{\prime }$ through Equation (6). When the current access satellite resources are insufficient, the ES chooses ${\bar{v}}^{\prime }$ as the access satellite. Because ${\bar{v}}^{\prime }$ is selected in the original path, no further routing is required after the ES switches to backup access satellite ${\bar{v}}^{\prime }$. The choice of ${\bar{v}}^{\prime }$ reduces the switching times and the consumption of resources by rerouting.

For the scenario in Figure 7, only access satellite $\bar{v}$ is in set $V^{\prime \prime }$. ES cannot obtain the status of satellite resources other than $\bar{v}$ through the ACKs and cannot select backup access satellite ${\bar{v}}^{\prime }$ in set $V^{\prime }$. To solve this problem, we perform the following optimization of the DAA algorithm. If $S_{n,m}^{L}$ is the next hop of $\bar{v}$ but $S_{n,m}^{L}\notin V^{\prime \prime }$, $S_{n,m}^{L}$ adds IP to the ACKs before forwarding the ACKs. After receiving the ACKs, the ES decodes the IP address of the next hop of $\bar{v}$ and uses this IP address as the virtual destination address. When $\bar{v}$ resources are insufficient, the ES selects ${\bar{v}}^{\prime }$ according to Figure 5, then adds the virtual destination address to the data packet to send to ${\bar{v}}^{\prime }$. ${\bar{v}}^{\prime }$ computes the route to the virtual destination address. The satellite corresponding to the virtual destination address receives the packet forwarded by ${\bar{v}}^{\prime }$ and forwards the packet according to the previous path. As the distance from ${\bar{v}}^{\prime }$ to the virtual destination address is smaller than that to the actual destination address, the selection and use of virtual destination addresses can reduce the resource cost of routing after switching to a backup access satellite.

#### 3.2.2. ESL Interruption or $\bar{v}$ Failure

ESL interruptions or $\bar{v}$ failures can cause the ES to fail to send packets to the satellite and fail to receive the ACKs forwarded by $\bar{v}$. This paper determines whether the fault mentioned above by acknowledging the timeout and setting the timeout timer T [28]:

(8) $$\begin{array}{cc} T=4\left[(1-\kappa )T+\kappa RTT\right] & 0\leq \kappa \leq 1 \end{array},$$

where RTT denotes Round Trip Time, and κ denotes the smoothing factor. T is started after the ES sends the data packet. The ES adopts the same strategy as Section 3.2.1 to select ${\bar{v}}^{\prime }$. When T=0, the ES switches ESL to ${\bar{v}}^{\prime }$. The DAA algorithm pseudo code is shown in Algorithm 1.
**Algorithm 1** Dynamic domain-based adaptive access algorithm (DAA)**DAA in ES****Require:** Orbital elements: $\left[i,\Omega ,e,\omega ,a,M_{0}\right]$, Time: *t*, Minimum elevation angle: σ1:The satellites in the communication domain are determined by Equations (1)–(3);2:The coverage time of the satellites in the communication domain is calculated by Equation (3);3:**if**$t_{V}^{con}\neq \emptyset$**then**4:    Equation (6) is used to calculate the first time access satellite of ES;5:**else**6:    Calculate access satellites set Q through the flow chart shown in Figure 5;7:**end if**8:ES decodes the ACKs returned by $\bar{v}$;9:**if**$U_{C}<U_{C}^{T}$ OR $U_{E}<U_{E}^{T}$ **then**10:   Switch the ESL to the ${\bar{v}}^{\prime }$ according to Section 3.2.111:**end if**12:**if**T<0**then**13:    Switch the ESL to the ${\bar{v}}^{\prime }$ according to Section 3.2.214:**end if****DAA in**$S_{n,m}^{L}$**Require:** Location of the ES: $\left[LON_{ES},LAT_{ES}\right]$, Maximum communication distance of ES: $D_{max}$, Time: *t*1:**if**$S_{n,m}^{L}$ is $\bar{v}$ **then**2:    $S_{n,m}^{L}$ as the access satellite, add $\left[LON_{ES},LAT_{ES},D_{max}\right]$ to the data packet;3:    $S_{n,m}^{L}$ add $\left[C_{L,n,m}^{t},E_{L,n,m}^{t}\right]$ to the ACKs;4:**else**5:  The satellites on the data packet forwarding path are judged whether in the communication domain of the ES by Equation (3);6:     **if** $S_{n,m}^{L}\in V^{\prime \prime }$ **then**7:          $S_{n,m}^{L}$ add $\left[C_{L,n,m}^{t},E_{L,n,m}^{t}\right]$ to the ACKs; 8:     **else if** $S_{n,m}^{L}\in V^{\prime }$ and $S_{n,m}^{L}\notin V^{\prime \prime }$ **then**9:          $S_{n,m}^{L}$ deletes $\left[LON_{ES},LAT_{ES},D_{max}\right]$ from the data packet; 10:   **else if** $S_{n,m}^{L}\in V^{\prime }$ and $S_{n,m}^{L}\notin V^{\prime \prime }$ and $S_{n,m}^{L}$ is the next hop of $\bar{v}$ **then**11:         Add the IP address of $S_{n,m}^{L}$ to the ACKs;12:   **end if**13:**end if**

In the algorithm, DAA in ES is the pseudo-code of the DAA algorithm running in ES, and DAA in $S_{n,m}^{L}$ is the pseudo-code of the DAA algorithm running in the satellites. Lines 1–7 of the DAA in ES are used to determine the set of satellites for the first access by ES. This process does not require ES to interact with the satellites. Lines 8–14 are used to complete the ESL switching when the current access satellite has insufficient resources or failure. The prior knowledge to complete the process is obtained through the ACKs without additional packet transmission. DAA in $S_{n,m}^{L}$ mainly provides prior knowledge for selecting backup access satellites by ES.

## 4. Experimental Verifications

To verify the effectiveness of the DAA algorithm, the first phase of the StarLink constellation is designed in this paper using Satellite Tool Kit (STK) [29]. The DAA algorithm is verified and analyzed by MATLAB based on the satellites’ track data derived from STK. The DAA algorithm is compared with the access selection algorithm based on a single parameter [6,10] and the access selection algorithm based on multi-attribute [18].

### 4.1. Simulation Parameters

**Figure 8:**
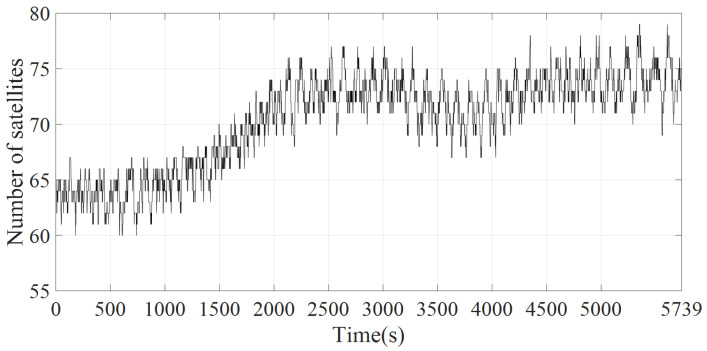
The number of satellites accessible to $ES_{1}$ varies over time.

**Figure 9:**
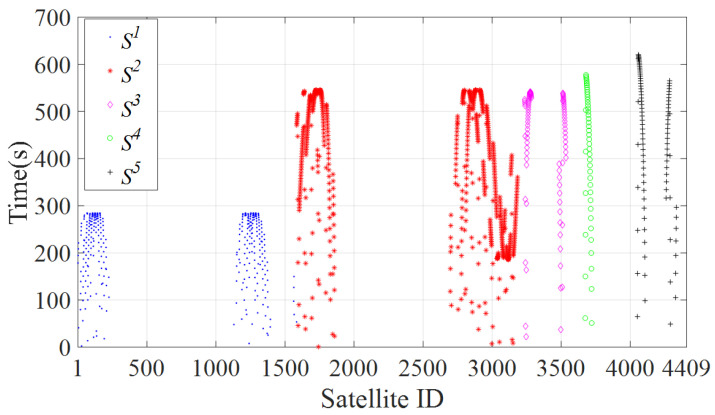
Coverage time of satellites.

The constellation topology used in the simulation is shown in Figure 1. The ESs selected in the simulation are all located in Beijing, China. The ESs parameters are shown in Table 2. The number of satellites that can be accessed by $ES_{1}$ during one orbital cycle of $S^{1}$ varies with time, as shown in Figure 8. The orbital period of $S^{1}$ is *T*:

(9) $$T=2\pi \sqrt{{\left(R+H_{1}\right)}^{3}/GM}$$

where *R* is the earth’s radius, $H_{1}$ is the height of $S^{1}$ from the ground, and GM is the gravitational coefficient of the earth. Based on Equation (9), the orbital period T≈ 5739 s of $S^{1}$ is obtained by STK. The satellite coverage time in each shell during *T* is shown in Figure 9. $ES_{1}$, $ES_{2}$, $ES_{3}$, and $ES_{4}$ are denoted by $ES_{1–4}$. As the Poisson model can well simulate network traffic [30], the $t_{u}$ values of $ES_{1–4}$ are set to obey the Poisson distribution of λ = 500 s, λ = 800 s, λ = 1000 s, and λ = 1500 s, respectively. From the satellite coverage time of each shell in Figure 9, when $t_{u}$ obeys the Poisson distribution of λ = 500 s, $ES_{1}$ can complete data upload without switching access satellite in most cases. When $t_{u}$ obeys the Poisson distribution of λ = 800 s and λ = 1000 s, $ES_{1}$ must switch once access satellite to complete data upload in most cases. When $t_{u}$ obeys the Poisson distribution of λ = 1500 s, $ES_{1}$ must switch twice to access satellite to complete data upload in most cases.

### 4.2. Simulation Analysis

**Figure 10:**
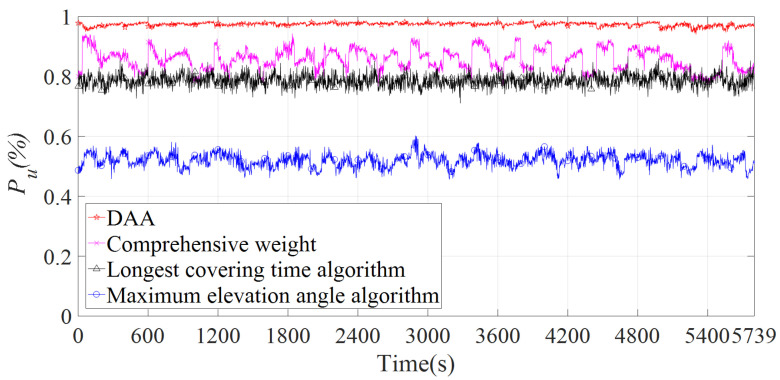
Coverage time utilization with different access algorithms.

In the scenario without insufficient resources, satellite failures, and ESLs interruption, the relationship between the $P_{u}$ value and the access time of the DAA algorithm and the traditional access algorithm is shown in Figure 10. The vertical coordinates in the graph indicate the average value of $P_{u}$ obtained from multiple experiments. The horizontal coordinate indicates the moment when $ES_{1–4}$ start transmitting packets. The simulation results show that the average $P_{u}$ values of the DAA algorithm, comprehensive weighting algorithm, longest covering time algorithm, and maximum elevation angle algorithm are 97%, 85%, 79%, and 51%. Therefore, the coverage time of the access satellite selected by the DAA algorithm is the most matched with $t_{u}$. The $P_{u}$ value of the DAA algorithm is the most stable because each access uses the process shown in Figure 5 to select the satellites with the most matching $t_{u}$ as the access satellites.

**Figure 11:**
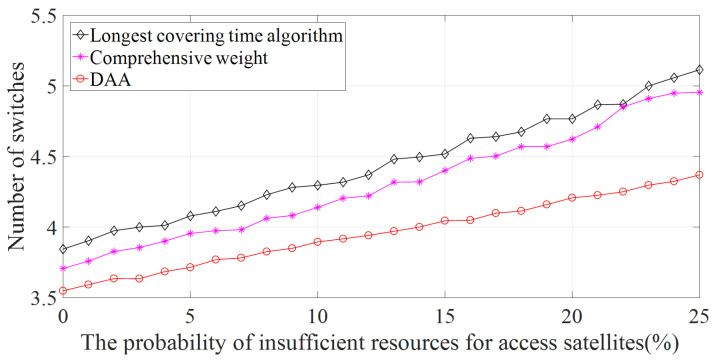
Relationship between the probability of insufficient resources for access satellites and switching times.

The relationship between the probability of insufficient satellite resources and switching times is shown in Figure 11. The number of switches in the figure is the average of the experimental results obtained from multiple experiments. The vertical coordinate is the sum of the number of switches required by $ES_{1–4}$ to complete the data transmission. The horizontal coordinates are the probability of insufficient satellite resources. As the maximum elevation angle access algorithm has the minimum $P_{u}$ value, the switching times are much more than the other three algorithms. Therefore, the experimental results of the switching times of the maximum elevation angle access algorithm are no longer displayed in Figure 11.

It can be seen from Figure 11 that the average switching times of the DAA algorithm under different probabilities are less than those of the other two algorithms; this is because the $P_{u}$ value of the DAA algorithm is larger than the traditional access algorithm. In addition, the DAA algorithm can further reduce the switching times by obtaining the resource status of some satellites in the communication domain by ACKs. In the simulation process, compared with the traditional access algorithm, the DAA algorithm does not need the interaction information between ES and the satellites before selecting the access satellite, and the maximum $t_{u}$ value can be achieved only through the calculation of ES. The DAA algorithm saves channel resources and reduces the cost of ES and satellites.

## 5. Conclusions

The DAA algorithm proposed in this paper provides a new way of thinking to solve the access problem of large-scale satellite networks. First, we dynamically update satellites in the communication domain of the ES in real-time. Then, the set of access satellites is determined based on the relationship between the $t_{u}$ values and the satellite coverage time in the communication domain. Finally, to solve the problem of insufficient satellite resources, access satellite failure, or ESLs interruption, we propose solutions for different scenarios. Through theoretical analysis, the use of the virtual destination address in the DAA algorithm and the strategy of selecting access satellites in the original path can reduce the rerouting overhead. Experimental results show that the DAA algorithm achieves maximum $P_{u}$ compared to traditional access algorithms. As the probability of insufficient satellite resources increases, the DAA algorithm has the least switching times compared to traditional access algorithms. Therefore, the DAA algorithm is more suitable for large-scale satellite networks than traditional access algorithms.

With the development of satellite Internet, the design of routing algorithms for large-scale satellite networks will be promising research. Based on this work, we will study routing algorithms for large-scale satellite networks suitable for the DAA algorithm in the future. where not only the $P_{u}$ value and switching times but also other aspects will be considered. For example, the impact of the DAA algorithm on end-to-end delay, throughput, and robustness of satellite networks.

## Figures and Tables

**Table 1:** Shells configuration for the first phase of StarLink constellation deployment.

Shell	Orbits	Satellites/Orbit	H (Km)	Inclination
$S^{1}$	72	22	550	53°
$S^{2}$	32	50	1110	53.8°
$S^{3}$	5	80	1130	74°
$S^{4}$	5	75	1275	81°
$S^{5}$	6	75	1325	70°

**Table 2:** Earth stations parameters.

ID	Latitude	Longitude	Altitude (Km)	Elevation
$ES_{1}$	39.908	116.420	0.049	25°
$ES_{2}$	40.117	116.228	0.038	25°
$ES_{3}$	40.072	116.257	0.041	25°
$ES_{4}$	40.558	116.976	0.151	25°

## Data Availability

Not applicable.

## References

[1] Del Portillo I., Cameron B.G., Crawley E.F. (2019). A technical comparison of three low earth orbit satellite constellation systems to provide global broadband. Acta Astronaut..

[2] Kuang L., Chen X., Jiang C., Zhang H., Wu S. (2017). Radio Resource Management in Future Terrestrial-Satellite Communication Networks. IEEE Wirel. Commun..

[3] Chaudhry A.U., Yanikomeroglu H. (2021). Laser Intersatellite Links in a Starlink Constellation: A Classification and Analysis. IEEE Veh. Technol. Mag..

[4] Sedin J., Feltrin L., Lin X. Throughput and Capacity Evaluation of 5G New Radio Non-Terrestrial Networks with LEO Satellites. Proceedings of the GLOBECOM 2020—2020 IEEE Global Communications Conference.

[5] Liu Y., Liao C. (2016). The development of emerging satellite internet constellations. Sci. Technol. Rev..

[6] Yao Y., Liang X.W. (2013). Dynamic routing technique based on LEO and GEO double-layered satellite network. Syst. Eng. Electron..

[7] Zhou J., Sun L., Han C., Guo X., Wang J. (2017). Channel resources management strategy for multi-layer satellite network. Syst. Eng. Electron..

[8] Iuoras N., Le-Ngoc T. (2005). Dynamic capacity allocation for quality-of-service support in IP-based satellite networks. IEEE Wirel. Commun..

[9] Park H., Yoon S., Kim T., Park J., Do M., Lee J. Vertical handoff procedure and algorithm between IEEE802.11 WLAN and CDMA cellular network. Proceedings of the 7th CDMA International Conference (CIC 2002).

[10] Ling X., Hu J.H., Wu S.Q. (2000). Access Schemes for LEO Satellite Mobile Communication Systems. Acta Electron. Sin..

[11] Shirata S., Yamaoka K. Reducing total call-blocking rates by flow admission control based on equality of heterogeneous traffic. Proceedings of the 13th International Telecommunications Network Strategy and Planning Symposium.

[12] Xu L., Wang P., Li Q., Jiang Y. (2017). Call Admission Control with Inter-Network Cooperation for Cognitive Heterogeneous Networks. IEEE Trans. Wirel. Commun..

[13] Al-Gizawi T., Peppas K., Axiotis D.I., Protonotarios E.N., Lazarakis F. (2005). Interoperability criteria, mechanisms, and evaluation of system performance for transparently interoperating WLAN and UMTS-HSDPA networks. IEEE Netw..

[14] Majlesi A., Khalaj B.H. An Adaptive Fuzzy Logic Based Handoff Algorithm For Interworking Between Wlans And Mobile Networks. Proceedings of the 13th IEEE International Symposium on Personal, Indoor and Mobile Radio Communications.

[15] Chen J.Z., Liu L.X., Hu X.H. (2013). Towards an end-to-end delay analysis of LEO satellite networks for seamless ubiquitous access. Sci. China—Inf. Sci..

[16] Gopal R., Gopal R. (2011). Cross-plane information sharing for QoS in satellite–terrestrial integrated packet networks. Int. J. Satell. Commun. Netw..

[17] Lu Y., Chen Y., Huang S., Zhang M. (2021). An Access Network Selection Algorithm for Terrestrial-Satellite Networks Based on a QoS Guarantee. Math. Probl. Eng..

[18] Zhou W.B. (2018). Research of Resource Management and Access Technology Based on Satellite Network. Master’s Thesis.

[19] He Q., Xiang Z., Ren P. (2021). A CLSTM and transfer learning based CFDAMA strategy in satellite communication networks. PLoS ONE.

[20] Lee S., Lee M., Lim S.J. (2018). A Virtual Allocation Based Dynamic Assigned Multiple Access Scheme in Multi-Frequency TDMA Satellite Networks. J. Korean Inst. Commun. Inf. Sci..

[21] Zhou X., Jia S., She Y. (2005). Delay performance of the improved CFDAMA MAC protocol via satellite. J. Nanjing Univ. Sci. Technol..

[22] LeNgoc T., Krishnamurthy S.V. (1996). Performance of combined free/demand assignment multiple-access schemes in satellite communications. Int. J. Satell. Commun..

[23] Kassing S., Bhattacherjee D., Aguas A.B., Saethre J.E., Singla A. Exploring the “Internet from space” with Hypatia. Proceedings of the IMC’20: ACM Internet Measurement Conference.

[24] Chen Q., Giambene G., Yang L., Fan C., Chen X. (2021). Analysis of Inter-Satellite Link Paths for LEO Mega-Constellation Networks. IEEE Trans. Veh. Technol..

[25] Dai G., Chen X., Zuo M., Peng L., Wang M., Song Z. (2018). The Influence of Orbital Element Error on Satellite Coverage Calculation. Int. J. Aerosp. Eng..

[26] Han C., Huo L., Tong X., Wang H., Liu X. (2020). Spatial Anti-Jamming Scheme for Internet of Satellites Based on the Deep Reinforcement Learning and Stackelberg Game. IEEE Trans. Veh. Technol..

[27] Osoro O.B., Oughton E.J. (2021). A Techno-Economic Framework for Satellite Networks Applied to Low Earth Orbit Constellations: Assessing Starlink, OneWeb and Kuiper. IEEE Access.

[28] Loukili A., Wijesinha A.L., Karne R.K., Tsetse A.K. TCP’s Retransmission Timer and the Minimum RTO. Proceedings of the 21st International Conference on Computer Communications and Networks (ICCCN).

[29] Zhang R., Xu S., Chen Z. Multi-target flight scene simulation based on STK/X. Proceedings of the 2nd Advanced Information Technology, Electronic and Automation Control Conference (IAEAC).

[30] Karagiannis T., Molle M., Faloutsos M., Broido A. A nonstationary Poisson view of Internet traffic. Proceedings of the IEEE INFOCOM 2004.

